# Differential expression of PLAC1 and Netrin-1 in liver metastasis of colorectal cancer and its predictive value

**DOI:** 10.1186/s12876-023-02908-y

**Published:** 2023-08-11

**Authors:** Zhijun Wang, Lei Deng, Xiwen Xu, Lianwu Zhao

**Affiliations:** 1 Department of Blood Transfusion, Gaoxin Branch of the First Affiliated Hospital of Nanchang University, Nanchang, 330029 Jiangxi P.R. China; 2 General Department of oncology, The Second Affiliated Hospital of Nanchang Medical College, Jiangxi Cancer Hospital, Jiangxi Clinical Research Center for Cancer, Nanchang, 330029 Jiangxi P.R. China; 3 Department of Gastroenterology, Gaoxin Branch of the First Affiliated Hospital of Nanchang University, No. 7889, Changdong Avenue, Gaoxin district, Nanchang City, 330029 Jiangxi Province P.R. China

**Keywords:** PLAC1, Netrin-1, Colorectal cancer, Liver metastasis, Differential expression, Prediction model

## Abstract

**Objective:**

To explore the differential expression of placental specific gene 1 (PLAC1) and neurite guidance factor 1 (netrin-1) in colorectal cancer (CRC) liver metastasis and its predictive value.

**Methods:**

Paraffin specimens of primary CRC were selected, including 60 simple colorectal cancer specimens and 47 liver metastasis specimens. At the same time, 40 cases of normal colorectal mucosa were taken as the control group. The expression of PLAC1 and Netrin-1 in tissue was detected by immunohistochemistry (IHC). The correlation between PLAC1 and Netrin-1 expression and clinicopathological characteristics of patients with CRC liver metastases was analyzed. Logistic analysis was adopted to analyze the influencing factors of liver metastasis in CRC. A prediction model was established and ROC curve was used to detect the discrimination of the prediction model. The clinical value of PLAC1 and netrin-1 in predicting liver metastasis of CRC was analyzed using ROC curve. The relationship between the expression of PLAC1 and netrin-1 and the prognosis of CRC patients with liver metastasis was analyzed using Kaplan Meier survival curve.

**Results:**

The positive staining of PLAC1 and netrin-1 was mainly located in the cytoplasm by IHC detection. Positive expression of PLAC1 and netrin-1 in CRC tissues was markedly higher than that in normal colorectal mucosal epithelium (*P* < 0.05). Positive expression of PLAC1 in metastatic group was higher than that in non-metastatic group without significant difference (*P* > 0.05). The metastasis group had much higher positive expression of netrin-1 than the non-metastasis group (*P* < 0.05). The content of PLAC1 in the tissues of CRC with liver metastasis had a close relationship with differentiation degree and lymph node metastasis (*P* < 0.05). The expression of Netrin-1 in the tissues of CRC with liver metastasis was associated with Dukes stage, differentiation degree and lymph node metastasis (*P* < 0.05). Logistic regression analysis showed that Dukes stage, differentiation, lymph node metastasis, CEA, Alb and D-dimer were the independent risk factors for liver metastasis of CRC (*P* < 0.05). The model was constructed according to the regression coefficients and constant terms, and the discrimination of the prediction model was evaluated using ROC curve, with the AUC of 0.903 (95% CI 0.831 ~ 0.975), the sensitivity of 93.80%, the specificity of 80.00%, and the Jordan index of 0.738. The AUC of PLAC1 and netrin-1 alone and combined detection to predict liver metastasis of CRC were 0.805, 0.793 and 0.921, respectively. The survival time of patients with positive PLAC1 and netrin-1 expression were sharply shorter than that of the patients with negative expression (*P* < 0.05).

**Conclusions:**

The expression of PLAC1 and netrin-1 was strongly increased in CRC with liver metastasis, which had a certain clinical value in predicting liver metastasis of CRC. Dukes stage, differentiation degree, lymph node metastasis, CEA, Alb and D-dimer were independent risk factors for liver metastasis of CRC, and the model based on these indicators had good discrimination for effectively evaluating the risk of liver metastasis in CRC.

## Introduction

Colorectal cancer (CRC) is a malignant tumor with the highest incidence rate and mortality in the world, the incidence rate of which is gradually increasing with the change of people’s living habits and the increasing social pressure [1]. The pathogenesis of CRC is complex, and the early onset is relatively hidden. Surgery combined with radiotherapy and chemotherapy is still the main treatment for CRC. For patients in advanced stage, the prognosis of patients is still poor even after effective treatment. Tumor invasion and metastasis is an important reason for the development of patients’ condition [2]. Many studies have found that [3], liver is an important organ for CRC to metastasize, and liver metastasis is also one of the main causes of death. Therefore, it is important to find an effective method for early diagnosis and timely intervention of CRC patients with liver metastasis to improve the survival time and survival rate of patients.

Placenta specific gene 1 (PLAC1) is a member of the Cancer/Testis Antigen (CTA) family, which is restrictively expressed in placental trophoblast cells. Previous studies found that [4, 5], abnormal expression of PLAC1 was found in nasopharyngeal carcinoma, cervical cancer and other malignant tumor tissues, and its expression level was closely related to poor prognosis of patients. Chen et al. [5] found that PLAC1 promoted the proliferation, metastasis and invasion of cervical cancer cells by activating the epithelial mesenchymal transition, and high expression of PLAC1 was considered to be a marker of poor prognosis in cervical cancer patients. Yu et al. [6] found that PLAC1 was significantly highly expressed in osteosarcoma cells, and promoted the development of osteosarcoma by enhancing cell proliferation, invasion and metastasis. Neurite guidance factor 1 (Netrin-1) is a secreted protein with similar structure to laminin. Research showed that [7] Netrin-1 participated in the regulation of angiogenesis, cell migration, cell apoptosis and other biological processes. Villanueva et al. [8] found that Netrin-1 promoted neuroblastoma metastasis in immunodeficient mouse models. El-Gamal et al. [9] found that Netrin-1 was highly expressed in bladder cancer tissues and was involved in the process of muscle invasion and metastasis of bladder cancer, which could be used as a predictor of local recurrence and metastasis. However, it is unclear whether PLAC1 and Netrin-1 are involved in the process of liver metastasis of CRC.

In this study, the expression difference of PLAC1 and Netrin-1 in liver metastasis of CRC was detected and a prediction model was built, aiming to provide an important basis for clinicians to evaluate patients’ condition.

## Materials and methods

### General materials

**Fig. 1:**
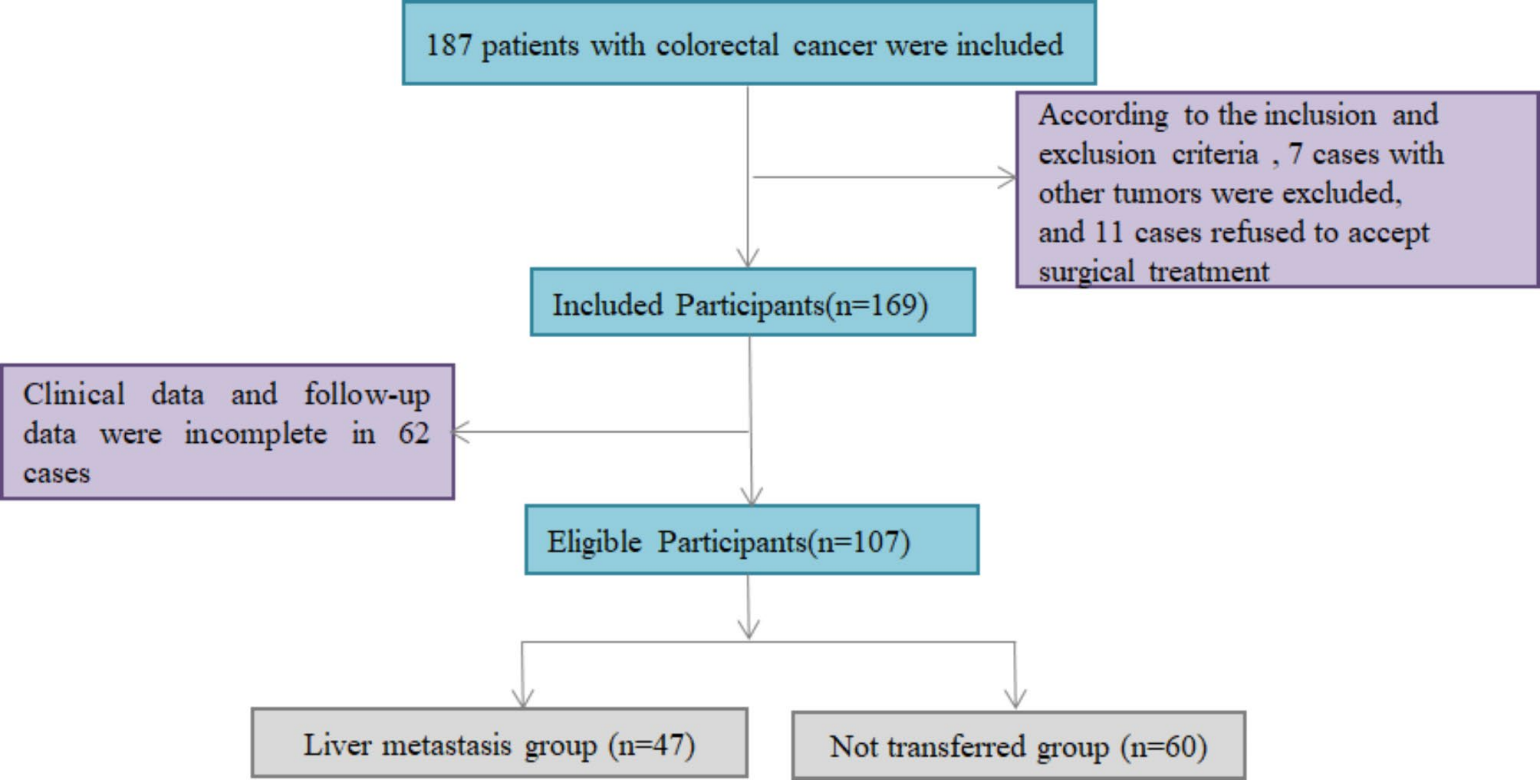
The selection process of general data

A total of 107 patients who underwent surgical resection of primary CRC in our hospital during January 2016 to January 2017 were picked as the study subjects. According to whether there existed liver metastasis, there existed 60 cases of simple CRC tissue and 47 cases of CRC liver metastasis ones, all of which were obtained by surgery and pathology. The selection process of general materials was shown in Fig. 1. Inclusion criteria: (1) All patients were definitely diagnosed as CRC by pathology (Hematoxylin eosin staining methods) [10]. (2) All patients diagnosed as liver metastasis met the clinical diagnostic criteria established by the French Society of Medicine and Surgery [11] and were confirmed by pathology or typical imaging. (3) Patients aged over 18 years old. (4) All patients did not receive any adjuvant treatment before operation. (5) All patients had complete clinical data and postoperative follow-up data. (6) Both patients and their families informed consent to participate in this study. Exclusion criteria: (1) Patients with liver metastasis caused by other tumors other than primary liver cancer or CRC. (2) Patients combined with other malignant tumors. (3) Patients who refused to accept surgical treatment. (4) Patients with severe liver and kidney dysfunction. (5) Patients who did not received serum tumor markers detection before operation; (6) Patients with incomplete clinical data and follow-up data. At the same time, 40 cases of normal colorectal mucosa were taken as the control group. The study was approved by the hospital Ethics Committee.

### Methods

Preparation process of tissue section [12]: The slide shall be soaked in the cleaning solution, fully cleaned and dried, and then treated with poly lysine (purchased in Shenzhen Zike Biotechnology Co., Ltd., Longgang District, Shenzhen, Guangdong Province, China, product No.: ZKP0791) for anti-stripping. Melted paraffin (purchased in Shanghai Guangrui Biotechnology Co., Ltd., Fengxian District, Shanghai, China) is made into 4.0 cm × 2.0 cm × 1 cm blank wax block. A 10 × 8 arranged organization array was designed, and the module was made using the drilling machine. The original wax was baked in a 45 ℃ oven for 5 ~ 10 min. The tissue slices are taken from the selected part of the tissue block with a drilling needle and placed into a pre designed array module to arrange the tissue slices. HE staining sections were made from donor tissues for morphological observation, and the required targets were accurately marked on the donor wax block. The target tissue was drilled with a perforator and transferred to the corresponding hole position of the receptor wax block, that is, the required array wax block was prepared. Tissues section with the thickness of 4–5 μm was made using conventional methods and was installed on the slide. Finally, the tissue section was placed face down on the copper plate. The module was gently pressed to make the tissue column horizontal in the module. The paraffin block was sectioned consecutively, and the tissue section was pasted on the slide for immunohistochemistry (IHC) experiment.

HIC staining [13]: The sections of CRC, liver metastatic CRC and normal intestinal mucosa were immunohistochemical stained with EnVision method. Phosphate buffer saline (PBS, purchased in Shanghai Fusheng Industrial Co., Ltd., Minhang District, Shanghai, China, product No.: A01 × 2515) was used as the primary antibody in the negative control group, and the known positive sections were used as the positive control. Positive particles of PLAC1 (PLAC1 polyclonal antibody was purchased from Shanghai Qunji Biotechnology Co., Ltd., product No.: PAB 19,325), Netrin-1 (Netrin-1 antibody was purchased from Shenzhen Haodi Huatuo Biotechnology Co., Ltd., product No.: PL 0402304) were located in the cytoplasm. Two representative high magnification visual fields were selected from each sample, 200 tumor cells were selected from each sample for counting, and the average value was taken. The number of positive cells < 5% was judged as negative (-), 5%~25% as weak positive (+), 25%~50% as moderate positive (++), and > 50% as strong positive (+++). Results were judged using double blind method and each section was counted by two pathologists.

### Data collection

The clinical data of selected cases were collected, including age, gender, maximum tumor diameter, Dukes stage, degree of differentiation, lymph node metastasis, histological type, serum carcinoembryonic antigen (CEA), serum carbohydrate antigen 199 (CA-199), D-dimer and albumin (Albumin, Alb). Logistic multivariate analysis was used to analyze the risk factors of liver metastasis in CRC patients.

### Statistical analysis

SPSS 22.0 data statistics software (IBM, Armonk, New York, USA) was used for calculation according to different observation indexes and data. The enumeration data was expressed in [cases (%)], and compared using χ ^2^ test. The measurement data were in accordance with the normal distribution through the normal distribution test, and were expressed in the form of ($\bar x \pm s$). Logistic multivariate regression analysis was used to analyze the risk factors of liver metastasis of CRC. ROC curve was used to analyze the clinical value of PLAC1 and Netrin-1 in predicting liver metastasis of CRC. Kaplan Meier survival curve was used to analyze the relationship between the expression of PLAC1, Netrin-1 and the prognosis of CRC patients with liver metastasis. *P* < 0.05 indicated that the difference was statistically significant.

## Results

### Expression of PLAC1 and Netrin-1 in tissues detected by IHC

**Fig. 2:**
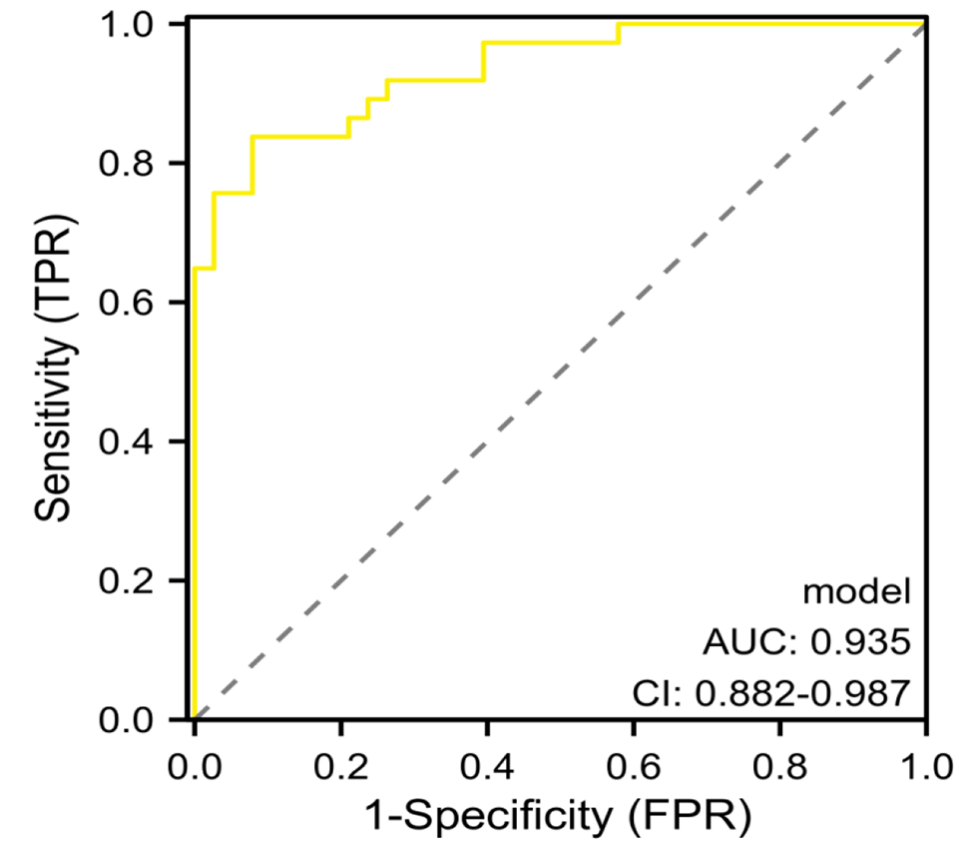
ROC curve of predictive model for liver metastasis of CRC

IHC detection showed that the positive staining of PLAC1 and netrin-1 was mainly located in the cytoplasm. Positive expression of PLAC1 and netrin-1 in CRC tissues was markedly higher than that in normal colorectal mucosal epithelium (*P* < 0.05). Positive expression of PLAC1 in metastatic group was higher than that in non-metastatic group without significant difference (*P* > 0.05). The metastasis group had much higher positive expression of netrin-1 than that in the non-metastasis group (*P* < 0.05, Table 1; Fig. 2). Thus, these results indicated that PLAC1 and Netrin-1 might be involved in the occurrence and development of CRC liver metastasis.

**Table 1:** Expression of PLAC1 and Netrin-1 in tissues detected by IHC [cases (%)]

Groups	Cases	PLAC1		Netrin-1	
		Positive	Negative	Positive	Negative
Control group	40	0 (0.00)	40 (100.00)	4 (10.00)	36 (90.00)
Non-metastatic group	60	34 (56.67)^a^	26 (43.33)	32 (53.33)^a^	28 (46.67)
Metastatic group	47	29 (61.70)^a^	18 (38.30)	37 (78.72)^ab^	10 (21.28)
*F*		41.488		41.372	
*P*		< 0.001		< 0.001	

Note: ^a^*P*<0.05 compared with the control group; ^b^*P*<0.05 compared with non-metastatic group

### Correlation between PLAC1, Netrin-1 and clinicopathological characteristics of CRC patients with liver metastasis

The CRC patients with liver metastasis (low differentiation and lymph node metastasis) had significantly higher positive expression of PLAC1 than these with moderately high differentiation and no lymph node metastasis (*P* < 0.05). Netrin-1 was significantly higher in CRC with liver metastasis who had Dukes stage C, low differentiation and lymph node metastasis than these with Dukes stage A + B, moderately high differentiation and no lymph node metastasis (*P* < 0.05, Table 2). These results further confirmed that PLAC1 and Netrin-1 were involved in the development of CRC liver metastasis, and were related to clinicopathological features.

**Table 2:** Correlation between PLAC1, Netrin-1 and clinicopathological characteristics of CRC patients with liver metastasis [cases (%)]

Groups		PLAC1		*χ* ^*2*^	P	Netrin-1		*χ* ^*2*^	P
		Positive 29	Negative18			Positive 37	Negative 10		
Gender (%)	Male	17 (58.62)	10 (55.56)	0.043	0.836	21 (56.76)	6 (60.00)	0.034	0.854
	Female	12 (41.38)	8 (44.44)			16 (43.24)	4 (40.00)		
Age (year)	< 60	14 (48.28)	7 (38.89)	0.396	0.529	22 (59.46)	6 (60.00)	0.001	0.975
	≥ 60	15 (51.72)	11 (61.11)			15 (40.54)	4 (40.00)		
Maximum tumor diameter (cm)	< 5	14 (48.28)	6 (33.33)	1.014	0.314	23 (62.16)	5 (50.00)	0.484	0.487
	≥ 5	15 (51.72)	12 (66.67)			14 (37.84)	5 (50.00)		
Dukes stage (%)	Stage A + B	19 (65.52)	11 (61.11)	0.093	0.760	9 (24.32)	6 (60.00)	4.611	0.032
	Stage C	10 (34.48)	7 (38.89)			28 (75.68)	4 (40.00)		
Differentiation degree (%)	Low	15 (51.72)	3 (16.67)	6.255	0.044	20 (54.05)	1 (10.00)	6.288	0.043
	Medium	12 (41.38)	14 (77.78)			14 (37.84)	7 (70.00)		
	High	2 (6.90)	1 (5.56)			3 (8.11)	2 (20.00)		
Lymph node metastasis (%)	Yes	19 (65.52)	6 (33.33)	4.620	0.032	25 (67.57)	3 (30.00)	4.613	0.032
	No	10 (34.48)	12 (66.67)			12 (32.43)	7 (70.00)		
Histological type (%)	Adenocarcinoma	19 (65.52)	10 (55.56)	0.479	0.787	26 (70.27)	4 (30.00)	4.114	0.128
	Mucinous adenocarcinoma	6 (20.69)	5 (27.78)			7 (18.92)	5 (60.00)		
	other	4 (13.79)	3 (16.67)			4 (10.81)	1 (10.00)		

### Univariate analysis of liver metastasis in CRC

Significant differences were observed in Dukes stage, degree of differentiation, lymph node metastasis, CEA, Alb and D-dimer between patients with liver metastasis and patients without liver metastasis (*P* < 0.05), but there existed no significant differences in gender, age, maximum tumor diameter, histological type and CA199 between two groups (*P* > 0.05, Table 3). These results suggested that the occurrence of liver metastasis of CRC was the result of the combination of various factors such as clinicopathological features.

**Table 3:** Univariate analysis of liver metastasis in CRC [(‾*x* ± *s*), cases (%)]

Factors		Metastatic group (n = 47)	Non-metastatic group (n = 60)	*t/χ* ^*2*^	*P*
Gender	Male	30 (63.83)	34 (56.67)	0.563	0.453
	Female	17 (36.17)	26 (43.33)		
Age (year)	< 60	19 (40.43)	20 (33.33)	0.572	0.449
	≥ 60	28 (59.57)	40 (66.67)		
Maximum tumor diameter (cm)	< 5	16 (34.04)	34 (56.67)	1.258	0.262
	≥ 5	31 (65.96)	26 (43.33)		
Dukes stage	Stage A + B	16 (34.04)	33 (55.00)	4.663	0.031
	Stage C	31 (65.96)	27 (45.00)		
Degree of differentiation	Low	25 (53.19)	14 (23.33)	12.838	0.002
	Medium	15 (31.91)	21 (35.00)		
	High	7 (14.89)	25 (41.67)		
Lymph node metastasis	Yes	44 (93.62)	25 (41.67)	31.058	< 0.001
	None	3 (6.38)	35 (58.33)		
Histological type	Adenocarcinoma	37 (78.72)	53 (88.33)	4.267	0.118
	Mucinous adenocarcinoma	8 (17.02)	3 (5.00)		
	other	2 (4.26)	4 (6.67)		
CEA (ng/ml)		7.16 ± 2.28	6.25 ± 1.81	2.302	0.023
CA199 (kU/L)		42.17 ± 12.96	39.64 ± 12.09	1.041	0.300
Alb (g/L)		27.85 ± 4.81	31.58 ± 5.36	6.301	< 0.001
D-dimer (μg/L)		188.25 ± 46.29	169.20 ± 22.65	2.792	0.006

### Logistic multivariate regression analysis of risk factors for liver metastasis of CRC

The assignment of each factor was shown in Table 4. Logistic regression analysis showed that Dukes stage, differentiation degree, lymph node metastasis, CEA, Alb, D-dimer, and positive expression of PLAC1 and Netrin-1 were the independent risk factors for liver metastasis of CRC (*P* < 0.05, Table 5), indicating that positive expression of PLAC1 and Netrin-1 and clinicopathological features jointly promoted the occurrence of liver metastasis of colorectal cancer.

**Table 4:** Variable assignment

Variate		Assignment
X1	Dukes stage	0 = A + B stage, 1 = C stage
X2	Degree of differentiation	0 = high, 1 = medium, 2 = low
X3	Lymph node metastasis	0 = none, 1 = yes
X4	CEA	0=<6.85 ng/ml, 1 = ≥ 6.85 ng/ml
X5	Alb	0=>29.33 g/L, 1 = ≤ 29.33
X6	D-dimer	0=<178.69 μg/L, 1 = ≥ 178.69 μg/L
X7	PLAC1	0 = negative, 1 = positive
X8	Netrin-1	0 = negative, 1 = positive
Y	Live metastasis	0 = unhappen, 1 = happen

**Table 5:** Logistic multivariate regression analysis of risk factors for liver metastasis of CRC

Indicators	B value	Standard error	Wald value	*P* value	*OR* value	95% CI	
						Lower limit	Upper limit
Dukes stage	0.960	0.417	5.042	0.016	2.611	1.129	6.037
Degree of differentiation	1.283	0.431	8.590	0.003	3.650	1.525	6.561
Lymph node metastasis	1.315	0.471	7.573	0.007	3.754	1.455	9.672
CEA	0.942	0.416	5.223	0.021	2.572	1.136	5.767
Alb	1.170	0.452	6.356	0.012	3.270	1.288	7.253
D-dimer	1.882	0.572	11.064	0.001	6.660	2.172	18.525
PLAC1 positive expression	0.538	0.214	6.320	0.012	1.713	1.126	2.605
Netrin-1 positive expression	1.396	0.511	7.463	0.007	4.039	1.484	10.996
Constant	0.959	0.417	5.042	0.021	2.621	—	—

### Establishment of predictive model for liver metastasis of colorectal cancer

The predictive model was constructed according to the regression coefficients and constant terms in the Table 5. The equation of the prediction model was Logit (P) = 0.960 × Dukes stage (0 represented Phase C, 1 represented Phase B, and 2 represents Phase A) + 1.283 × Differentiation degree (0 indicated high differentiation, 1 indicated medium differentiation, and 2 indicated low differentiation) + 1.315 × Lymph node metastasis (0 meant yes, and 1 meant no) + 0.942 × CEA (actual value) + 1.170 × Alb (actual value) + 1.882 × D-dimer (actual value) + 0.538 × PLAC1 (0 meant negative, and 1 meant positive) + 1.396 × Netrin-1 (0 meant negative, and 1 meant positive) + 0.959. With the predictive probability value as the test variable and whether liver metastasis occurred as the state variable, the ROC curve was used to evaluate the discrimination of the prediction model. The results showed that the area under the curve (AUC) was 0.935 (95% CI 0.882 ~ 0.987), the sensitivity was 85.29%, the specificity was 92.11%, and the Yoden index was 0.774 (Fig. 2). These results demonstrated that this prediction model has certain clinical application value, and could be used as a prediction tool for the occurrence of liver metastasis in CRC, and guide timely clinical intervention and treatment.

### ROC curve analysis of the clinical value of PLAC1 and Netrin-1 in predicting liver metastasis of CRC

**Fig. 3:**
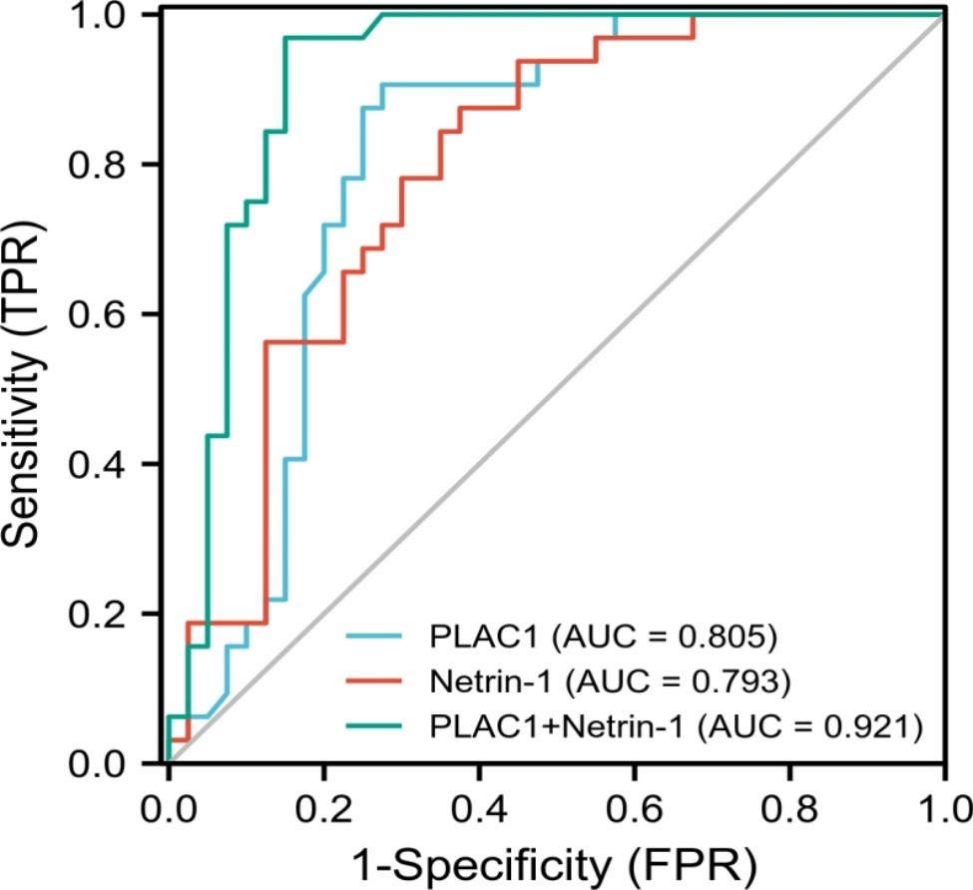
ROC curve analysis

ROC curve showed that the AUC of PLAC1 and netrin-1 alone and combined detection to predict liver metastasis of CRC were 0.805, 0.793 and 0.921, respectively. Therefore, both alone and combined detection of the two indicators had certain clinical value, and the combined detection had higher predictive value (Table 6; Fig. 3). These results suggested that the diagnostic efficiency of PLAC1 and Netrin-1 could be further improved based on the combined detection method.

**Table 6:** ROC curve analysis of the clinical value of PLAC1 and Netrin-1 in predicting liver metastasis of CRC

Variable	AUC	Sensitivity (%)	Specificity (%)	Positive predictive value (%)	Negative predictive value (%)	Yoden index	*P* value	*95% CI*
PLAC1	0.805	90.60	72.50	72.50	90.60	0.631	< 0.001	0.704 ~ 0.906
Netrin-1	0.793	87.50	62.50	65.10	86.20	0.500	< 0.001	0.675 ~ 0.911
Combined detection	0.921	96.90	85.00	83.80	97.10	0.819	< 0.001	0.831 ~ 1.011

### KM survival curve analysis about the relationship between the expression of PLAC1, Netrin-1 and the prognosis of CRC patients with liver metastasis

**Fig. 4:**
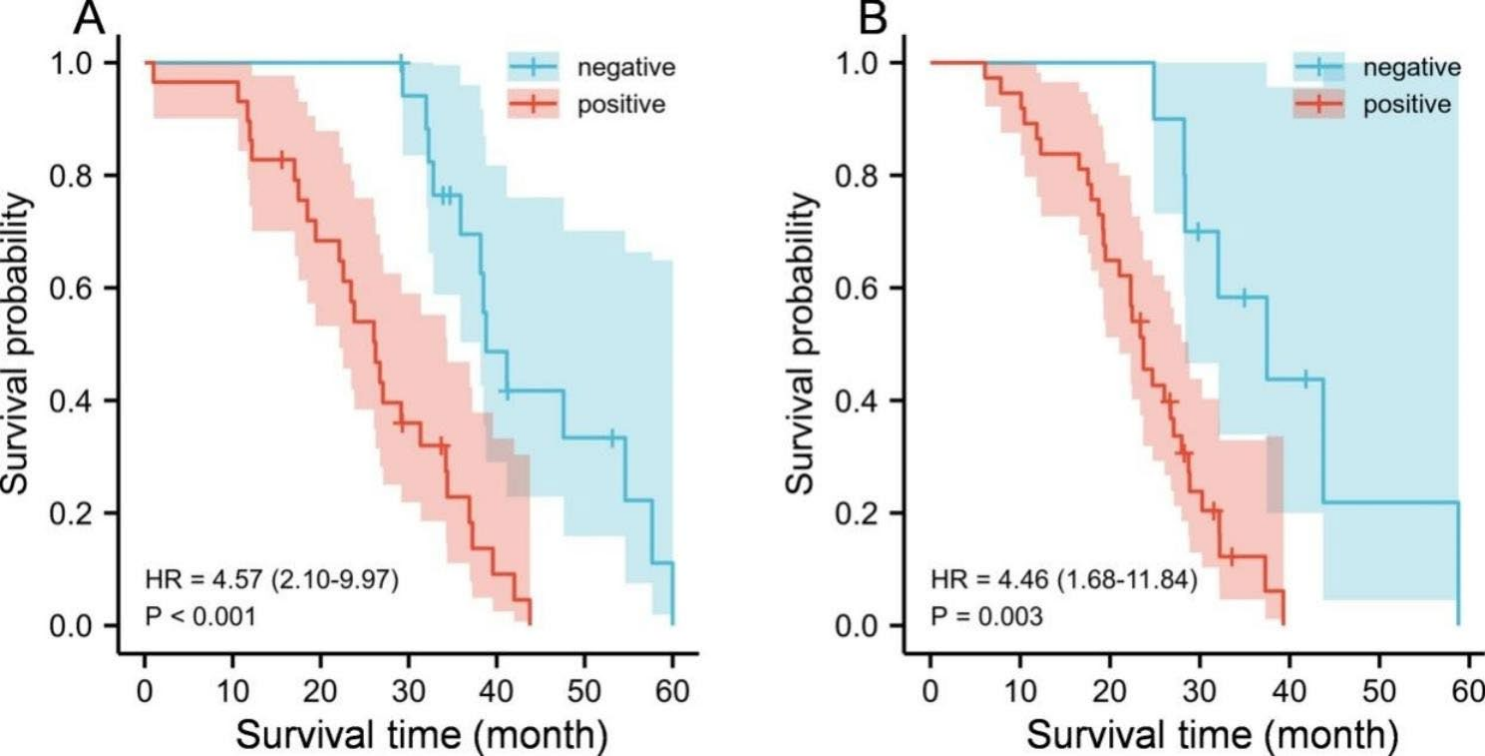
KM survival curve of CRC patients with liver metastasis. **A**: Survival curve of patients with different PLAC1 expression; **B**: Survival curve of patients with different Netrin-1 expression

A total of 47 patients with liver metastasis in CRC were followed up for 60 months with a 5-year survival rate of 17.02% (8/47). The median survival period of patients with positive and negative PLAC1 expression was 25 months and 39 months, respectively. The median survival period of patients with positive and negative Netrin-1 expression was 23 months and 35 months, respectively. The survival time of patients with positive PLAC1 and netrin-1 expression were sharply shorter than the patients with negative expression (*P* < 0.05, Fig. 4). These results demonstrated that PLAC1 and Netrin-1 could not only be used as biomarkers to predict liver metastasis in CRC, but also can be used to evaluate the prognosis of patients.

## Discussion

Colorectal cancer (CRC) is one of the most common malignant tumors with complex pathogenesis in clinic. At present, it is believed that the occurrence and development of CRC may be the result of multiple factors. CRC has a high incidence rate and mortality rate and poor prognosis, which seriously affects the quality of life and even life health of patients [14]. With the continuous progress of medical technology, the early diagnosis rate of CRC has been largely improved. However, the 5-year survival rate of patients of CRC is still low, and the postoperative mortality is still high [15]. The liver is the main location of distant metastasis of CRC, and the occurrence of liver metastasis is an important reason affecting the prognosis of patients, and there is no radical treatment method at present [16]. It is important to find out the influencing factors of liver metastasis of colorectal cancer in order to build a predictive model for early diagnosis and treatment to effectively improve the prognosis of patients.

PLAC1 is a placental specific gene. Under normal circumstances, PLAC1 is mainly expressed in placenta and testis, and the expression level of PLAC1 is strongly increased when tumors occur [17]. Some scholars found in the study of prostate cancer [18] that PLAC1 has high reactivity in prostate cancer cells, and antibody-drug conjugates based on anti PLAC1 may pave the way for the development of more reliable, efficient and new immunotherapy for prostate cancer patients. It is reported [19] that the high expression of PLAC1 in colon cancer can be used as a predictor of poor prognosis, and it may enhance the potential of liver metastasis by activating PI3K/Akt/NF kB signaling pathway. This is similar to the results of this study that the CRC patients with liver metastasis had higher positive expression of PLAC1 than the patients without liver metastasis. Netrin-1 is a soluble neurite guidance factor that can bind to immunoglobulin like transmembrane receptors to exert a related role [7]. The study found that [20], the level of serum Netrin-1 in CRC patients was strongly higher than that in healthy subjects, and the risk of CRC in patients with high expression of Netrin-1 was also markedly increased. The results of this study showed that patients in metastatic group had much higher positive expression of Netrin-1 than the patients in non-metastatic group and in normal colorectal epithelial mucosa group, indicating that PLAC1 and Netrin-1 had the value of screening CRC patients with high liver metastasis tendency, played a key role in promoting tumor growth, invasion and distant metastasis in tumor progression, and had the potential of new tumor markers. In addition, ROC curve analysis showed that PLAC1 and Netrin-1 were of great value in predicting liver metastasis of CRC. PLAC1 is expressed in nucleus in the early stage of CRC, and trophoblast cells invade the endometrium accompanied with the formation of blood vessels during embryo implantation, which is very similar to the growth, migration, and invasion of CRC, and both trophoblast cells and CRC tumor cells express PLAC1 [19]. The expression of PLAC1 in patients with CRC acts on oncogene loci to achieve the regulation of tumor genes, and the more obvious the expression in patients with low tumor tissue differentiation, the more it can be regulated by PLAC1 [21]. Netrin-1 interacts with neo-genin (neonatal protein), enhances the chemotaxis of CD4 + T cells, and triggers a pro-inflammatory response, playing a key role in the regulation of the tumor microenvironment [22]. Netrin-1 promotes proliferation, migration, and adhesion of vascular endothelial cells and smooth muscle cells, and its pro-angiogenic effects may be necessary for tumor growth [23]. All these above results indicated that PLAC1 and Netrin-1 could be used as effective indicators for evaluating liver metastasis of CRC.

In the present study, Logistic regression analysis showed that Dukes stage, differentiation degree, lymph node metastasis, CEA, Alb, D-dimer, and positive expression of PLAC1 and Netrin-1 were the independent risk factors for liver metastasis of CRC, which indicated that positive expression of PLAC1 and Netrin-1 and clinicopathological features jointly promoted the occurrence of liver metastasis of colorectal cancer. The reason may be that Dukes staging is an important indicator of tumor biological behavior. With the postponement of Dukes staging, the lower the degree of tumor differentiation is, the higher the degree of malignancy is, the greater the risk of distant metastasis is, and the worse the prognosis of patients is. Previous studies have shown that [24], whether lymph node metastasis occurs is closely related to the prognosis of patients with malignant tumors, and metastasis is an important part of Dukes staging. The tumor spread far away through lymph nodes. It is reported that [25], compared with patients with negative lymph node metastasis in CRC, patients with positive lymph nodes have a worse prognosis. The biomarkers detection is an effective method to assess the development of patients’ condition and predict the prognosis of patients, including serological markers, oncology related genes, etc. CEA, Alb, D-dimer, etc. are the most commonly used related indicators. Previous study found that [26] CEA could enhance the metastatic potential of CRC through a variety of ways to protect metastatic cells from death. Alb is involved in maintaining the alternating osmotic pressure of blood and promoting the transport of vascular substances. Research shows that [27], patients with low Alb level have significantly increased protein catabolic capacity, but their synthetic capacity is reduced, which may cause malnutrition. Low Alb level may be an important indicator of poor prognosis. Previous study found that [28], D-dimer was an important indicator for early screening and auxiliary diagnosis of CRC, and the level of D-dimer was closely related to the malignancy of the disease. In addition, KM survival curve analysis showed that the positive expression of PLAC1 and Netrin-1 was significantly related to the poor prognosis of CRC patients with liver metastasis. PLAC1 is expressed in tumor tissues, but little or no expression in normal tissues. The expression of PLAC1 in CRC is conducive to targeted therapy for CRC, can also be applied to the diagnosis and treatment of CRC, and is more conducive to the prognosis of patients with CRC [29]. Netrin-1 can promote tumor cell proliferation, invasion and metastasis, and its receptor colon cancer deletion gene is one of the markers of the CRC progression and metastasis, thus it is speculated that Netrin-l may participate in the occurrence and development of CRC by regulating the expression and function of its receptor colon cancer deletion gene [30]. The results of this experiment further proved that as important active factors in promoting the invasion and metastasis of CRC, PLAC1 and Netrin-1 are closely related to the degree of tumor differentiation, are the key molecules to promote liver metastasis in patients, and are also the bottlenecks restricting the therapeutic effect of surgery. PLAC1 and Netrin-1 can become important markers for judging tumor metastasis, and can also be potential targets for the treatment of CRC.

In general, Dukes stage, differentiation degree, lymph node metastasis, CEA, Alb and D-dimer were independent risk factors for liver metastasis of CRC. The expression of PLAC1 and Netrin-1 in tissues of CRC with liver metastasis was largely increased, which have important value in predicting liver metastasis of CRC. However, due to the limited research time and the sample size included in the study, the mechanism of PLAC1 and Netrin-1 affecting liver metastasis of CRC is still unclear, which will be further explored in our follow-up research.

### Research significance

This study confirms that PLAC1 and Netrin-1 play an important role in liver metastasis of CRC, which makes it possible to detect some metastatic lesions hidden in the liver at an early stage and carry out terminal targeted treatment for such high-risk patients. This study proves that PLAC1 and Netrin-1 are related to the degree of differentiation and lymph node metastasis of CRC liver metastasis, and can better judge the possibility of liver metastasis, which has certain clinical research value. Through the combined detection method, the diagnostic efficiency of PLAC1 and Netrin-1 can be further improved, and the parallel test of PLAC1 and Netrin-1 can make the sensitivity reach 96.90 and the specificity increase to 85% when diagnosing early liver metastasis, which will be conducive to clinical judgment and has certain clinical application value. Applying the results of this study to the clinic can help the early detection of liver metastases and serve as a good indicator for targeted treatment of CRC with high metastatic tendency.

## Data Availability

The datasets used and/or analyzed during the current study are available from the corresponding author on reasonable request.
